# On the Local Treatment of Pulmonary Cavities

**Published:** 1874-06-01

**Authors:** F. Mosler

**Affiliations:** Greifswald


### ON THE LOCAL TREATMENT OF PULMONARY CAVITIES, BY PROF. F. MOSLER, OF GREIFSWALD.

### A Resume of the Author's Various Reports, by the Editor of the Allgemeine Wiener Med. Zeitung.

*Translated for* The Examiner *by* Dr. H. Gradle.

THE general, scientific and practical interest connected with this new mode of research; the, at least, partially favorable expectation of substituting another for our previous role of idle spectators in this terrible malady; the more active, dignified interference of the true physician, which hope has received a new foundation by employment of this novel therapeutic method, is certainly sufficient excuse for informing our readers of the last progress in this direction.

Since scientific research has shown that under certain conditions every pulmonary inflammation may bring about caseous degeneration and suppuration of the tissues it invades, and that the caseous matter is infectious, a more local treatment has seemed to be proper, for it is well known that the caseous processes are quite analogous to the morbid changes induced by contagion, and thus new *foci* of disease continue to arise, not only in the lungs, but in all organs, to which the secretions of the affected parts have access. Mosler has observed in his clinic a patient, suffering from caseous pneumonitis, who, in spite of repeated advice, made no efforts to throw out his sputa, but continued to swallow them, until his death by a secondary affection of the intestines. Mosler has now adopted the view that since disinfection reduces considerably, if not destroying totally, the inoculability of infectious substances, it is a clear therapeutic indication to render harmless and, if possible, remove from within, the caseous matter retained in the system. Acting accordingly, he has proposed to enter pulmonary cavities by a way through the thoracic walls, after inhalations of carbolic acid conjoined with the use of expectorants had been employed in many cases with but little show of success. The first attempt was made by the author on a consumptive patient, aged 51 years, more with the intention of testing the possibility of the method than as a mode of cure, as the last stage of the disease, which the patient had reached, promised but little chance of amelioration. The highly cachectic individual had a superficial cavity in the right upper lobe, traceable to the fourth rib.

On the first of November, 1872, Mosler introduced the rather stout canula of Tiersch's aspirator in the second intercostal space at a distance of six cm. from the right sternal margin, and pushing it in deeply, injected twenty minims of a highly diluted solution of potassic permaganate, whereupon the aspirator was removed, the canula being retained in the cavity in order to repeat the injection. On the fourth day the canula became obstructed and was removed also, all operations having been endured by the patient without any ill effects. The same procedure was repeated by Mosler in February, 1873, on another patient, with bronchiectatic cavity of the left side, the secretion of which had assumed a very foul smelling, putrid state. Five injections were well borne and resulted in an improved character of the sputa, as well as in several ameliorations. Thus convinced of the possibility of the method, Mosler adopted the modification to establish a complete drainage for the secretion. A painter, aged 49 years, treated for five years in the author's clinic for a cavity in the right upper lobe and who had had attacks of hemoptysis, was greatly emaciated, frequently in a febrile condition, and whose urine was highly albuminous from amyloid renal degeneration, was operated upon, July 2, 1873, by Profs. Hueter and Mosler, in the following way:

An incision three cm. in length was made along the upper margin of the third rib about five and a half cm. from the right sternal margin, separating the integument and superficial intercostal muscles. Now, since the long duration of the malady warranted the belief that firm adhesions existed between both pleural layers, the polypus-forceps was employed, after widening the wound, gradually encroaching upon the cavity, the opening of which was announced by a whistling inspiratory noise, while a foaming, purulent secretion escaped without any hemorrhage whatever. Dilating the aperture, a thick silver drainage-tube was inserted, attached with adhesive straps, and subsequently closed with carbolated lint, and the wound covered with the ice-bladder, the entire operation being well borne by the patient.

His temperature was registered that evening at 37.8°c (100°f,); the pulse beat eighty-four times per minute to thirty-six respirations; pus flowed freely through the canula, especially during a fit of coughing. The dressing was renewed repeatedly, while cough and expectoration gradually diminished. On the twelfth of July hemoptysis set in, probably caused by granulation in the cavity; soon, however, discontinuing on injection through the canula, of a diluted solution of liq. ferri perchlor.; subsequently by means of an atomizer the spray of a diluted mixture of carbolic acid and tincture of iodine was twice daily blown through the canula; feeling, as the patient professed, as if it entered the cavity. An injection of a larger quantity of potassic permanganate by means of Esmarch's irrigator was not as well borne, being followed by a sense of depression and febrile reaction; it was therefore discontinued, as a sufficient quantity of the atomized fluid could be introduced during deep respiratory exertions of the patient.

(*To be Continued.*)

